# Complexity of post-concussion syndrome assessment and management: a case for customizing rehabilitation

**DOI:** 10.1186/s12938-025-01380-x

**Published:** 2025-04-25

**Authors:** Nicholas Moser, Milos R. Popovic, Sukhvinder Kalsi-Ryan

**Affiliations:** 1 KITE Research Institute-University Health Network, Toronto, ON Canada; 2 Temerty Faculty of Medicine, Institute of Medical Science, University of Toronto, Toronto, ON Canada; 3 Institute of Biomedical Engineering, University of Toronto, Toronto, ON Canada; 4 Rehabilitation Sciences Institute, University of Toronto, Toronto, ON Canada; 5 Mechanical and Industrial Engineering, University of Toronto, Toronto, ON Canada

## Abstract

**Background:**

Post-concussion syndrome is a challenging condition to manage for even the most experienced chronic pain experts. Patients’ presentations are heterogeneous with symptoms spanning physical, cognitive and emotional domains. The symptoms reported are often non-specific, making it difficult for health professionals to prescribe effective rehabilitation. The aim of the present study was to examine the effectiveness of a customized rehabilitation program based on subgroup determination following a standardized clinical exam in adults with post-concussion syndrome.

**Methods:**

A total of 16 adults (mean age ± SD, 38.3 ± 12.5 years) with post-concussion syndrome participated in a 6-week rehabilitation program. Participants were recruited from external community concussion clinics around the greater Toronto area, Canada. Participants underwent a comprehensive standardized clinical exam to subgroup the ostensible symptom generators into either autonomic, cervical or vestibulo-ocular. Customized rehabilitation was then prescribed based on their subgroupings. The primary outcome measure was the Rivermead Post-Concussion Questionnaire (RPQ). Secondary outcome measures included the Patient Health Questionnaire-9 (PHQ-9), the Neck Disability Index (NDI), and exercise tolerance as assessed via the Buffalo Concussion Treadmill Test (BCTT).

**Results:**

Following 6 weeks of customized rehabilitation, participants on average experienced a significant and clinically meaningful change with respect to the RPQ-3 and RPQ-13 (*p* < 0.001). We also observed a significant change in all secondary outcome measures including a reduction in PHQ-9 (*p* < 0.01), NDI (*p* < 0.001) and exercise tolerance, expressed as heart rate threshold (*p* < 0.001).

**Conclusion:**

The standardized exam was feasible and useful in assisting the clinician in prescribing effective rehabilitation. The 6-week customized rehabilitation program demonstrated significant changes in patient-reported persistent post-concussion symptoms and exercise tolerance. The implementation of a customized program based on a standardized exam performed to subgroup the ostensible symptom generators may be key to successful management in this population.

## Background

Post-concussion syndrome (PCS) is a highly contested diagnosis [1] with much debate among clinicians and epidemiologists. Symptoms have been shown to be non-specific and the myriad of those reported can include physical complaints, such as headaches and dizziness; cognitive complaints, such as brain fog and impaired working memory; and affect/mood related issues, such as anxiety and depression as well as sleep impairment [2]. A main tenet to the debate is that there is currently no universally agreed upon definition for PCS with highly varied criteria between the current definitions used [3]. The heterogeneity of definitions used and criteria applied for clinical trials precludes the ability to perform meta-analyses and compare findings between trials. Ultimately this constrains our ability to generalize the findings and our ability to better understand the effects of rehabilitation on a larger scale.

Doubt surrounding PCS being a unique clinical entity largely stems from the current lack of organic injury unique to this condition or as the reliable cause for the reported symptoms. Preliminary evidence does indicate abnormal cerebral metabolism and perfusion [4] as well as electrophysiological recordings [5] in those with persistent symptoms. However, the few large-scale prognostic studies utilizing imaging modalities to identify these aforementioned impairments have limited utility given their small effects on outcomes [6]. There is also a lack of evidence comparing this population to an injured non-traumatic brain injury (TBI) control group [4, 7]. This is a necessary next step, given these populations have high rates of overlapping reported symptoms [1, 3]. Without an agreed upon reliable cause for the reported persistent symptoms a coherent evidence-based rehabilitative strategy remains enigmatic.

The implementation of reliable and objective diagnostic testing outside the acute setting has been largely absent. Currently there is a lack of valid and reliable outcome measures to assess persistent symptoms and their resolution following intervention. However, the Buffalo Concussion Treadmill Test (BCTT) and the widely utilized Rivermead Post-Concussion Questionnaire (RPQ) have been validated [8–10] and are two reliable tests that have utility in concussion studies [11, 12]. Regrettably, there is still no agreed upon RPQ score that would qualify as clinically relevant. Miller et al. [13] established a 15% cutoff for clinically meaningful changes in their clinical trial [13]. This cut-off score has been adopted by others [14] but it remains to be validated [15]. This is an important next step given observations made by Vikane et al., (2017) reporting that the RPQ appears to measure different features as the sum of scores differed in clinical significance from the total number of reported symptoms [14].

Despite contention regarding the diagnostic criteria and current lack of available biomarkers to confirm or refute the disorder, a significant minority of patients continue to report the persistence of symptoms following a concussive injury [16]. The most recent review found that one in three patients who present to the emergency department for a concussive injury will continue to report symptoms at the 6-month mark [17]. If it is not properly addressed this disorder can become problematic. Graff et al. [18] noted that 43% of patients who suffer a mild traumatic brain injury (mTBI) do not return to ordinary work 5 years post-injury [18]. In Ontario alone, the conservative estimated cost to medically manage this population was reported to be over $110 million annually [19]. Thus, there is a need to further evaluate more effective rehabilitation options.

Consistently higher quality of evidence is emerging for exercise and multimodal therapy; however, there is a need to further evaluate different therapies and refine exercise prescriptions (dose, frequency and intensity) given the varied responses and number of patients with persistent complaints despite therapy [20, 21]. Leddy et al. [22] proposed a systematic clinical examination to help the clinician identify one or more clinical profiles of the post-concussion patient in order to prescribe targeted therapies to optimize recovery [22]. This structured approach to concussion assessment and management is based on the notion that patients'symptoms emanate not only from the globally concussed brain, but also reflects dysfunction in sub-systems such as the cervical spine. By identifying the concussion subtypes, the rehabilitative approach can be customized to the patient and theoretically facilitate improved recovery.

The objective of this study was to define the effectiveness of a customized treatment program based on the findings of a standardized clinical exam performed to subgroup the ostensible persistent post-concussion symptom generators. More specifically, we report on the outcomes of a 6-week customized rehabilitation program in adults who suffer persistent post-concussion symptoms and have remained symptomatic despite having already undertaken rehabilitative care. This paper defines the results and also touches on the feasibility and clinical utility of the standardized exam to subgroup persistent post-concussion symptoms in adult patients for rehabilitation guidance.

## Results

### Participant demographics

A total of 16 adults participated in the study. All 16 participants that were enrolled completed the 6-week rehabilitation program. The rehabilitation participants received was customized based on the standardized exam performed at baseline. The intention of the standardized exam was to subgroup participants’ non-specific symptoms into ostensible symptom generators that the rehabilitation targeted.

No adverse events were reported by participants. As depicted in Table 1, the average age of the study population was 38 years with the majority of the participants (13 of 16) being female. There was a wide range of reported number of prior concussions (0–11) as well as symptom duration (1–8 months) prior to undertaking the rehabilitation program as part of this study. Only one participant reported undertaking additional treatment while in the trial, which encompassed occupational and vision therapy. They disclosed that they began these therapies before admittance to the trial. On average, participants demonstrated adherence with the rehabilitation recommendations, exceeding 80% (81.4 + 11) self-reported adherence to the recommended at-home exercises.

**Table 1:** Demographics and clinical characteristics (N = 16)

Years of age (mean ± SD, range)	38.3 ± 12.5, 21–61
Sex (male/female)	3/13
Race (White/Hispanic/Arab/Indian/Asian)	9/1/2/2/2
Number of prior concussions (mean ± SD, range)	2.1 ± 3, 0–11
Duration of symptoms, months (mean ± SD, range)	4.3 ± 1.9, 1–8
Mechanism of injury (MVA/sport/workplace/fall/other)	3/6/2/4/1
Baseline RPQ-3 score {out of 12} (mean ± SD, [95% CI])	7.1 ± 2.1, [6.1–8.2]
Baseline RPQ-13 score {out of 52} (mean ± SD, [95% CI])	27.4 ± 9.3, [22.8–31.9]
Baseline RPQ total symptom count {out of 16} (mean ± SD, [95% CI])	11.8 ± 3.6, [10.2–13.4]
Baseline PHQ-9 score {out of 27} (mean ± SD, [95% CI])	13.1 ± 5.5, [10.3–15.7]
Baseline NDI score {out of 50} (mean ± SD, [95% CI])	19.1 ± 5.8, [16.2–21.9]
Baseline BCTT result (percentage failed; mean heart rate BPM ± SD [95% CI]; heart rate range)	81%; 148.2 ± 23.8 [137.8–158.6]; 118–187

SD, standard deviation; CI, confidence interval; RPQ, Rivermead Post-Concussion Questionnaire; PHQ-9, Patient Health Questionnaire; NDI, Neck Disability Index; BPM, beats per minute; BCTT, Buffalo Concussion Treadmill Test

At baseline, defined as the point of enrollment, participants demonstrated a high symptom burden through their RPQ-3/13 questionnaire scores and the total number of RPQ symptoms reported. On average participants reported having moderate perceived depressive symptoms and moderate perceived neck disability as measured by the PHQ-9 and NDI, respectively (Table 1). As per the findings of the standardized exam, no participant demonstrated any hard neurological signs. All participants demonstrated an objective cervical spine impairment placing them into the cervical disorder subgroup. Thirteen of the participants had autonomic impairment as demonstrated by exercise intolerance and three of the 16 participants had an abnormal vestibular or oculomotor screen. Therefore, the majority of participants (13/16) demonstrated multiple subgroup impairments requiring multimodal therapies to address the various identified impairments (Table 2).

**Table 2:** Post-concussion symptom subgroup based on standardized clinical exam (N = 16)

Subgroup determination	Autonomic, cervical and vestibulo-ocular	Autonomic and cervical	Cervical
Number of participants	3	10	3

### Effects of customized rehabilitation on reported symptoms

Following 6 weeks of customized rehabilitation, participants made clinically meaningful changes in both the primary and secondary outcome measures (Table 3). On average participants’ RPQ-3 scores reduced from 7.1 (out of 12) at baseline to 2.5 exceeding the two-point change needed for MCID. On average, participants also lowered their RPQ-13 scores from 27.4 (out of 52) at baseline to 14.1 exceeding the MCID of eight points. The participants total symptom count reduced on average by 4.5 exceeding a significant change (*p* < 0.001).

**Table 3:** Overview of primary and secondary outcomes following 6-week rehabilitation

Primary and secondary outcome measures	Mean 6-week follow-up outcome measure scores	Mean change in outcome measure scores from baseline to 6-week follow-up	P-value
RPQ-3 score [out of 12] (mean ± SD, [95% CI])	2.5 ± 2, [1.7–3.5]	4.6 ± 2.4, [3.5–5.6]	p < 0.001
RPQ-13 score [out of 52] (mean ± SD, [95% CI])	14.1 ± 9.8, [9.8–18.4]	13.3 ± 8.4, [9.5–16.9]	p < 0.001
RPQ total symptom count [out of 52] (mean ± SD, [95% CI])	7.3 ± 4.3, [5.4–9.2]	4.5 ± 4, [2.7–6.3]	p < 0.001
PHQ-9 score [out of 27] (mean ± SD, [95% CI])	8.6 ± 4.5, [6.7–10.6]	4.4 ± 6, [1.7–7]	p < 0.01
NDI score [out of 50] (mean ± SD, [95% CI])	11.4 ± 7.3, [8.2–14.6]	7.8 ± 6.8, [4.7–10.7]	p < 0.001
BCTT result (percentage failed; heart rate mean BPM ± SD [95% CI]; heart rate range BPM)	18%; 176 ± 17.4, [168.4–183.6]; 145–195	27.8 ± 24.1, [10.6–38.4]	p < 0.001

SD, standard deviation; CI, confidence interval; RPQ, Rivermead Post-Concussion Questionnaire; PHQ-9, Patient Health Questionnaire; NDI, Neck Disability Index; BPM, beats per minute; BCTT, Buffalo Concussion Treadmill Test

With respect to the secondary outcomes, participants improved their baseline perceived self-rated depression scores as measured by the PHQ-9 from 13.1 (moderate depression) at baseline to an 8.6 (mild depression) at follow-up (*p* < 0.01). Participants improved their baseline perceived self-rated neck disability scores, as measured by the NDI, from 19.1 (moderate disability) at baseline to 11.4 (mild disability) at follow-up. Although a statistically significant change (*p* < 0.001) was achieved, the findings do not reflect a clinically meaningful change through the suggested MCID [44].

### Effects of customized rehabilitation on exercise tolerance

There was a considerable change in the number of participants with exercise intolerance following the 6-week customized rehabilitation. As depicted in Table 3, the number of participants who had exercise intolerance as measured by the BCTT decreased from 13 participants at baseline to three by the end of the program. This coincided with a statistically significant (*p* < 0.001) increase in participants' mean heart rate threshold, increasing from 148 BPM at baseline to 176 BPM at follow-up.

## Discussion

A 6-week customized rehabilitation program led to significant changes in self-reported symptoms and exercise tolerance in adults with persistent post-concussion symptoms who had previously limited response to therapy.

At the end of the trial only 18% of participants (*n* = 3 of 16) continued to demonstrate exercise intolerance. This was a significant (*p* > 0.001) change from baseline where over 80% of participants showed exercise intolerance. Participants significantly reduced their self-reported symptoms, dropping on average by 4.6 points on the RPQ-3 and by 13.3 points on the RPQ-13. This exceeded the established MCID of two points on the RPQ-3 and eight points on the RPQ-13. Furthermore, participants following the customized rehabilitation program reduced on average the total number of symptoms by 4.5. To the authors’ knowledge, there is currently no agreed upon change in the number of symptoms following intervention that would be deemed clinically meaningful. Tator et al. showed that each additional concussion symptom a patient experiences reduces the recovery rate by 25%. As such, future examination of effective rehabilitation should not solely focus on the impact of reducing symptom severity, but also on how rehabilitation can reduce the total number of symptoms reported [23].

Prior to the 6-week customized rehabilitation program, all 16 participants reported undertaking extensive rehabilitation that included physical therapies to the neck, balance exercises and advice to exercise aerobically. Despite this therapy, their self-reported symptoms had persisted on average for 4.5 months and were still rated as severe. Although participants had been directed to exercise, all but three had continued objective exercise intolerance.

Participants’ clinical presentations were heterogeneous. Specifically, at baseline participants endorsed on average 12 of 16 symptoms on the RPQ and following the standard examination various subgroup profiles were identified (Table 2). Complaints of persistent neck pain, sleep impairment and cognitive issues are common impairments in this population. Two commonly endorsed persistent symptoms, headaches and dizziness, have varied presentations, etiology and responses to therapies that drive uncertainty in the decisions attending health care practitioners make for their patients’ rehabilitation. As shown by the significant self-reported symptom changes post-rehabilitation, the standardized exam afforded the clinician to make a more informed decision regarding rehabilitative prescription despite the apparent complexity of this population's presentation.

The average time taken to complete the standardized examination and the primary and secondary outcome measures was 60 min. Almost all components of the exam can be done at any physical therapy office; however, a few pieces of equipment such as a treadmill, heart rate monitor and a laser pointer for neck proprioception testing are required and may not be present at all clinics. Although the majority of the tests in the exam continue to be reliant on patients' self-reported symptom exacerbation, the standardization of the clinical exam afforded a step-by-step approach to delineate non-specific symptom generators into ostensible subgroup(s) that were the presumed driver(s) for persistent impairments. Theoretically, this afforded the clinician with a more precise rehabilitation plan leading to improved patient-reported outcomes.

A consistent piece of feedback from the participants following the re-examination at 6 weeks was that the education regarding the subgroups and drivers of their persistent symptoms explained by the exam provided the needed reassurance for their prescribed rehabilitation. It is the opinion of the authors that the standardized exam is not only feasible regarding time for implementation but highly useful in directing patient care. Participant compliance is known to be low with rehabilitative recommendations, which likely has an impact on recovery. Given the high self-reported adherence to rehabilitative recommendations, the aforementioned education likely facilitated behavioral changes.

A major question that remains is why the participants responded well to a rehabilitation program despite previously undertaking therapies recommended by the current clinical guidelines [24] with no meaningful changes. As previously noted, at baseline 13 of the 16 participants continued to demonstrate exercise intolerance. By the end of the 6-week program that number dropped to three. As such, the answer to the question may reside in the differences in exercise form and intensity prescribed in the given rehabilitation program. In the customized program, participants’ exercise tolerance was re-examined weekly and their prescribed exercise training intensity of 85–90% below symptom threshold continued to be progressed along with their recovery. It is well documented that there is considerable variance in the response to a general exercise program [44–46] as measured by changes in muscle strength, size and cardiorespiratory fitness. The HERITAGE Family Study illustrated that regardless of age, sex, race and prior fitness status there are low, medium and high exercise “responders” [47]. The abolishment of exercise “non-responders” occurs when the exercise training dosage (time, frequency, volume and intensity) are manipulated, particularly the intensity [46, 49]. The progressive moderate-to-vigorous steady-state aerobic training participants undertook as part of this rehabilitation program may have been sufficiently intense to stimulate cardiovascular adaptations unlike the prior exercise training. Additional reasons for the observed changes in exercise tolerance exist and can include increased adherence compared to previous therapy programs they attempted given that participants were seen twice per week and self-reported greater than 80% engagement with the at-home rehabilitation recommendations.

This study has several strengths. Firstly, the demographics in this study make it among only a few rehabilitation trials that have examined a population outside young healthy high school or collegiate athletes [21]. Albeit the demographics were highly skewed to females, this is likely due to their documented increased risk of reporting persistent symptoms [23]. Secondly, all participants had undertaken prior therapy before being enrolled in the rehabilitation program. As such, the likelihood of regression to the mean and/or the placebo effect accounting for the changes observed is unlikely. Lastly, validated and reliable outcome measures were used to measure pre–post change scores.

This study also has several limitations. Firstly, although we observed significant and clinically meaningful changes in the primary and most secondary outcome measures assessed, we were limited to a 6-week follow-up period. No longer term follow-up was initiated. Secondly, the present study did not include an active control group to compare the customized rehabilitation program to. Thirdly, the present study had a limited sample size and the population incorporated was predominantly female and of White background limiting the generalizability of the findings. Lastly, given the limited sample size, a comparison across the differing modes/subgroups of treatments was not made, which may have affected their response as some participants received a “higher dose” of rehabilitation given they were placed into more than one subgroup.

As previously noted, there is a high cost associated with managing persistent post-concussion symptoms. A part of this cost is the large number of health service visits, which likely reflects the difficulty that patients experience in trying to find support for their symptoms. Given the documented prevalence of this disorder and cost for management, it is imperative that we continue to study and refine our rehabilitation prescription. These efforts could lead to a reduction in duration and frequency of visits to medical specialists and excessively long treatment plans with allied healthcare professionals.

The present study supports the use for a standardized exam, inclusive of exercise tolerance testing, as recommended by Matuszak et al. [25]. In a short period of time, the customized rehabilitation program demonstrated significant changes to patient-reported persistent post-concussion symptoms and exercise tolerance. The results provide preliminary evidence for superior patient-reported outcomes with a customized rehabilitation model that is based on subgrouping non-specific symptoms into ostensible symptom generators rather than the current symptom-based treatment approach.

## Methods

### Ethics approval

Ethics approval was granted by the University Health Network Research Ethics Board (#22–5560). All participants were required to provide both verbal and written informed consent before commencing any experimental procedures.

### Study design

We conducted a 6-week prospective cohort trial. Following the standardized examination and the completion of the primary and secondary outcome measures, participants were placed into one of three possible subgroups (autonomic, cervical or vestibulo-ocular) [22]. Participants’ customized care was dependent on the subgroup they were classified into. After 6 weeks of rehabilitation, participants underwent the end-point examinations.

### Population

A consecutive enrollment of 16 participants was obtained at the Toronto Rehabilitation Institute. 32 participants with post-concussion syndrome were screened in 2022–2023 for eligibility. Participants were recruited from external community concussion clinics around the greater Toronto area (eight clinics) as well as by internal advertisement at the University Health Network. Participants underwent therapy at the KITE Innovations and Rehabilitation Clinics located within the Toronto Rehabilitation Institute, Toronto, Ontario, Canada.

### Inclusion criteria

To be eligible, participants had to be 21 years and older; meet the definition of post-concussion syndrome as defined by Tator et al. [23], which requires a patient to report any three symptoms or more (from an inclusive list of the 40 most commonly reported persisting symptoms) lasting at least one month following the diagnosis of a concussion [23]. This definition was selected given the known limitations of the ICD-10, the fact that DSM-IV is no longer in practice and these previous definitions are biased towards those who are vulnerable to concussions or more severe forms of post-concussion syndrome [23]. Concussion was defined according to the 6th International Consensus Statement on Concussion in Sport [24]; have adequate language skills in English to read and take part in rehabilitation treatment programs; and demonstrate an objective impairment on the baseline standardized exam, placing the patient into one of the three subgroups.

### Exclusion criteria

Potential study participants were excluded should their clinical examination have been unremarkable for any positive physical findings and therefore their dominant symptomatology placed them into the affective/cognition subgroup described by Leddy et al. [22]. This exclusion was applied because we were interested in understanding the effects of a tailored physical rehabilitation program. As such, should the participants have not demonstrated any physical impairment, no physical rehabilitative interventions could be recommended. Additional exclusion criteria included chronic infectious disease; uncontrolled hypertension; other neurological disorders (not attributed to their primary diagnosis); cancer treatment (other than basal cell carcinoma), craniotomy, refractory subdural hematoma; long‐term use of psychoactive medications that would compromise their ability to comprehend and perform study activities; those with pacemakers or elevated cardiovascular risk; ongoing litigation surrounding their injury; those diagnosed with a moderate or severe brain injury prior to enrollment, or their persistent post-concussive symptoms had persisted beyond 12 months. Over the course of recovery there is a shift in the domain of symptom reporting with a reduction in physical symptoms and a stable or increase in cognitive and emotional/mood related symptoms [16]. As such, we applied this time restriction.

16 of the 32 participants screened were ineligible due to the duration of their symptoms exceeding one year (*n* = 11), had no positive examination findings to justify placement into one of the three subgroups (*n* = 3), or they could not comply with frequency of care (*n* = 2).

### Assessments/procedures

Participants underwent a comprehensive standardized clinical examination at the KITE Innovations and Rehabilitation Clinics to differentiate their post-concussive subgroup(s) [22]. Evaluation consisted of the recommended elements of a standardized clinical physical examination outlined by Matuszak et al. (2016) [25]. Specifically, the study coordinator (NM), a licensed chiropractor, performed all components of the examination including a neurological exam consisting of cranial nerve screen, motor testing of the upper and lower extremities and deep tendon reflexes; a musculoskeletal examination assessing for tenderness over the head and neck, range of motion of the cervical spine and Spurling test; joint position sense error test (JPSE) of the cervical spine in flexion, extension, lateral flexion and rotation, which has shown to be a reliable and relevant measure for the evaluation of neck pain [26–28]; balance/coordination examination assessing static and dynamic balance via the Balance Error Scoring System (BESS) and tandem walking with eyes open and closed [29]. The BESS consists of three static stances performed on both a firm and foam surface. The BESS has been shown to have moderate-to-good reliability in assessing static balance as well as has been shown to detect balance deficits in participants with concussion [30]. Continuing, a vestibular-ocular examination consisting of evaluation of the eyes in eight positions, evaluating nystagmus, saccades, smooth pursuit and near point convergence/accommodation. More tests were included if dizziness or imbalance were present: orthostatic vital signs via supine-to-stand stress test; Dix–Hallpike maneuver and assessment of dynamic visual acuity [31].

In addition to the examination outlined by Matuszak et al. [25], a physical exertion test was performed via the Buffalo Concussion Treadmill Test (BCTT) protocol to specifically examine exercise intolerance [9].

### Subgroup determination

As per the recommended elements of a standardized clinical physical examination outlined by Matuszak et al. in [25], a positive examination for the subgroups is outlined below.

A positive autonomic disorder screen, placing the patient into the autonomic subgroup, was defined as an inability to exercise at an age-appropriate heart rate threshold due to the exacerbation of concussion symptoms [9]. Exacerbation of symptoms was defined as an increase of three points or more of their reported symptoms from baseline during exercise on an 11-point numerical rating scale (0–10). When participants could exert themselves and achieve near their age-appropriate maximum heart rate without exacerbation of symptoms, then the etiology or justification for persisting concussion symptoms was ascribed to alternative problems [22].

A positive cervical spine screen, placing the patient into the cervical subgroup, was defined as having an abnormal cervical spine range of motion with pain as assessed by visual inspection by NM along with palpatory findings of facet joint restrictions and pain with supine joint motion palpation, cervical myofascial trigger points, or abnormal joint position error testing [26–28].

Finally, a positive vestibular or oculomotor screen, placing the patient into the vestibulo-ocular subgroup, was defined as an abnormal vestibulo-oculomotor screen (VOMS) [32], abnormal static and dynamic balance testing as assessed via the Balance Error Scoring System [30] and tandem walking [29], respectively, or when clinically indicated to perform, an abnormal vestibular special testing, such as Dix–Hallpike maneuver [31].

### Intervention

The study coordinator (NM), a licensed chiropractor with extensive experience in assessing and managing chronic pain patients, performed all components of the listed assessments/procedures and therapy at the KITE Innovations and Rehabilitation Clinics.

### Customized care program

During the first therapy session, participants were provided reassurance and psychoeducation via the bio-psycho-social model on understanding persistent symptoms [20, 21]. Participants were afforded two treatments per week over the course of 6 weeks. Thus, participants were provided 12 treatments over the course of the 6 weeks. The time frame of 6 weeks was selected given the treatment structure of prior clinical trials examining the effectiveness of physical interventions to address physical symptoms such as headaches, dizziness and neck pain [33]. Given the heterogeneity of symptoms, components of the treatments were customized to the participants based on what subgroup they were classified into at the baseline examination. Customized rehabilitative care differentiated by subgroups is outlined below:

- ● **Autonomic group—**participants received 30 min of supervised progressive sub-symptom aerobic exercise, as well as 20 min of mindfulness-based training twice per week. Sub-symptom aerobic exercise training was defined as any form of aerobic exercise the participants were able to perform at a prescribed target heart rate without causing a greater than a three-point change in total symptoms as rated on an 11-point pain numerical rating scale (0–10). The prescribed target heart rate was determined by the BCTT. Their target heart rate was 85–90% of their heart rate level when they failed the BCTT. This test was done weekly to ensure the timely advancement of the target heart rate while participants’ rehabilitation and recovery progressed. Participants were also instructed on various mindfulness-based techniques they could perform at home; however, in the clinic twice per week, a box-breathing technique was utilized. Outside the supervised rehabilitation sessions, participants were instructed to perform 30 min of sub-symptom aerobic exercise three times per week [20, 21] as well as 20 min of mindfulness-based training daily [34].
- ● **Cervical group—**participants received bi-weekly physical therapy to the cervical spine. Physical therapy included soft tissue therapy directed to the cervical myofascial tissues and graded cervical spine facet mobilizations and/or high velocity low amplitude cervical manipulation. Participants also received supervised 20 min of progressive neck isotonic strengthening exercises. They were also instructed to perform 20 min of general neck stretches, range of motion exercises and neck strengthening exercises twice a week outside the supervised sessions [20, 21].
- ● **Vestibulo-ocular group—**participants received bi-weekly customized oculomotor, vestibular and balance exercises including adaptation exercises, gaze stability exercises, visual–vestibular integration exercises, habituation exercises and static and dynamic balance exercises. They were also instructed to perform daily 15–20 min of customized vestibular and oculomotor exercises based on their clinical exam outside the supervised sessions [20, 21].

In cases where participants demonstrated multiple impairments on the standardized examination they received the listed treatments provided to the subgroups under which they were classified. However, the frequency (twice per week), duration (6 weeks) and length of care (60 min per session) did not change regardless of subgroup determination.

Lastly, participants were asked to refrain from seeking additional therapy. If they undertook additional therapy outside of the trial, they were asked to document the modality and frequency of said therapy in the provided treatment adherence calendar. The adherence calendar was given to all participants at the start of the trial in order to assist them with logging their daily rehabilitation. At the end of the 6 weeks, participants returned the calendar and their adherence to the rehabilitation was examined.

### Outcomes

#### Primary outcome

Participants’ overall symptomatology was evaluated with the Rivermead Post-Concussion Symptoms Questionnaire (RPQ). The RPQ is a subjective questionnaire composed of 16 concussion symptoms rated by patients according to their severity. For each symptom, patients rate the severity on a 0–4 point scale. The symptoms are categorized as physical, cognitive, and emotional. It has been demonstrated that when the entire 16-item questionnaire is summated together there is a poor overall fit to the Rasch model, suggesting all 16 items do not tap into the same underlying construct [10]. Therefore, the questionnaire should be split into two; the RPQ-3, which scales early post-concussion symptoms (headaches, dizziness and nausea) and the RPQ-13, which scales symptoms associated with having a greater impact on participation, psychosocial functioning and lifestyle. The RPQ-3 is scored 0–12 and the RPQ-13 is scored 0–52, with a higher score representing a greater impact [11]. The RPQ-3 has shown moderate test–retest reliability and the RPQ-13 has shown good test–retest reliability, with each forming a unidimensional construct for patients with head injury at three months post-injury [10, 11].

#### Secondary outcomes

The secondary outcome measures included the Patient Health Questionnaire-9 (PHQ-9), the Neck Disability Index (NDI), and exercise tolerance.

### Patient health questionnaire-9 (PHQ-9)

There is preliminary evidence to show that the presence of depression is a poor prognostic factor for recovery in patients with persistent concussion symptoms [35]. As such, a screening form for depression severity was measured via the PHQ-9. The PHQ-9 is a 9-item questionnaire. It is a multipurpose instrument for screening, measuring and monitoring the severity of perceived depression. The tool rates the frequency of the symptoms, which factors into the scoring of the severity index with a higher score representing a greater perceived symptom severity ranging from 0 to 27. The PHQ-9 score is interpreted as 0–4, minimal depression; 5–9, mild depression; 10–14, moderate; 15–19, moderately severe depression; 20–27, severe depression [36–39]. The PHQ-9 has been shown to have adequate reliability and good validity for the concussion population as well as the spinal pain population [36–39].

### Neck disability index (NDI)

Neck-related disability was measured via the NDI. The NDI is a 10-item questionnaire, which examines the impact of self-reported neck pain on various activities of daily living. The responses are summed for a total ranging from 0 to 50 with a higher score representing a more severe perceived disability. Benchmark interpretations have been suggested to include: 0–4, no disability; 5–14, mild disability; 15–24, moderate disability; 25–34, severe disability; and scores above 35, complete disability [40]. The questionnaire has demonstrated reliability, construct validity and responsiveness to change in various populations [40].

### Exercise tolerance

Exercise tolerance was assessed via the BCTT. The BCTT is a well-established aerobic exercise test designed to assess exercise tolerance, specifically to assess a patient's heart rate threshold [9]. BCTT has shown clinical utility in identifying those likely to suffer persistent symptoms and the test is an excellent guide for exercise prescription following a concussion [12, 41, 42]. Intolerance to exercise was defined as a failed BCTT. As defined by Leddy et al. [9], this was said to occur when a patient was unable to tolerate exercise at a heart rate threshold predicted for one’s age due to the exacerbation of their persistent concussion symptoms [9]. We specifically examined the change in heart rate threshold at baseline and at the 6 week follow-up.

The primary and secondary outcome measures were examined at participants' enrollment (baseline) and following the 6 weeks of rehabilitation (trial end-point).

### Statistical analysis

Descriptive statistics were applied to all outcome measures to assist in summarizing the results. To examine for change in the primary and secondary outcome measure post-intervention, statistical inferences were applied using a paired t-test and parameter estimations with a significance level of *p* < 0.05.

The primary outcome is a change in symptoms following rehabilitative interventions using the RPQ divided into the RPQ-3 and RPQ-13. Previous research examining the internal construct validity of the Rivermead Post-Concussion Symptom Questionnaire suggested that a rating of two or higher on at least three of the total 16 symptoms represents an unfavorable outcome [43]. As previously noted, unfortunately to date no validated change scores exist, thus we prescribed a cut-off of 15% improvement as minimal clinically important difference (MCID). This translates to a change of two points on the RPQ-3 and a change of eight points on the RPQ-13. This agrees with prior clinical literature examining changes post-intervention on concussion symptoms [13].

All unintended effects, harms and/or dropouts during the program were recorded.

## Data Availability

The used dataset can be made available upon request from the corresponding author.

## Abbreviations

RPQ: Rivermead post-concussion questionnaire
MCID: Minimum clinically important difference
PHQ-9: Patient health questionnaire
NDI: Neck disability index
TBI: Traumatic brain injury
mTBI: Mild traumatic brain injury
BCTT: Buffalo concussion treadmill test
